# Effect of obstructive sleep apnoea on retinal microvascular function: a randomised controlled trial

**DOI:** 10.1007/s00417-022-05596-8

**Published:** 2022-02-24

**Authors:** Chris D. Turnbull, James A. Stockley, Shyam Madathil, Syed S. A. Huq, Brendan G. Cooper, Asad Ali, Simon Wharton, John R. Stradling, Rebekka Heitmar

**Affiliations:** 1 Nuffield Department of Medicine, University of Oxford, Oxford, UK; 2 NIHR Biomedical Research Centre, University of Oxford, Oxford, UK; 3 Lung Function & Sleep, Queen Elizabeth Hospital, University Hospitals Birmingham NHSFT, Birmingham, B15 2GW West Midlands UK; 4 Department of Sleep and Respiratory Medicine, University Hospital Coventry and Warwickshire, Coventry, CV2 2DX Warwickshire UK; 5 Sleep Department, Heartlands Hospital, University Hospitals Birmingham NHSFT, Birmingham, B15 2GW West Midlands UK; 6 School of Applied Sciences, Department of Optometry and Vision Sciences, University of Huddersfield, Huddersfield, UK

**Keywords:** Obstructive sleep apnoea, Retinal vascular reactivity, Endothelial function, Autoregulation, Ocular vascular disease

## Abstract

**Purpose:**

Retinal microvascular endothelial dysfunction is thought to be of importance in the development of ocular vascular diseases. Obstructive sleep apnoea (OSA) causes macrovascular endothelial dysfunction, but the effect of OSA on retinal microvascular endothelial function is not known. We aimed to determine the effect of OSA on retinal microvascular function.

**Methods:**

We conducted a multi-centre, double-blind, randomised, parallel, controlled trial in patients with known moderate-to-severe OSA, established on continuous positive airway pressure (CPAP). Participants were randomised to 14 nights of either continued CPAP or sham CPAP to generate a return of OSA. Retinal vascular responses to flickering light were measured using dynamic vessel analysis both at baseline and after 14 nights of intervention. The primary outcome was the change from baseline to follow-up in the area under the curve of the arteriolar response to flickering light, sham CPAP versus continued CPAP.

**Results:**

Nineteen patients were randomised to sham CPAP, and 18 patients were randomised to continued CPAP. There was no significant effect of CPAP withdrawal and return of OSA on retinal responses, with a change in the area under the curve of the arteriole response to flickering light of + 3.8 arbitrary units (95% CI − 10.6 to + 18.2, *p* = 0.59), sham CPAP versus continued CPAP.

**Conclusions:**

CPAP withdrawal and a return of OSA had no significant effect on retinal microvascular responses. This contrasts with the effect of CPAP withdrawal on macrovascular endothelial function and suggests that OSA has different effects on macrovascular and microvascular endothelial function.

ISRCTN 78082983, 23/10/2014, Prospectively registered.
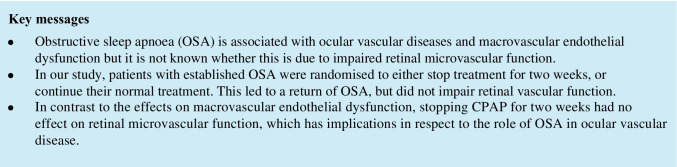

**Supplementary Information:**

The online version contains supplementary material available at 10.1007/s00417-022-05596-8.

## Introduction

Obstructive sleep apnoea (OSA) causes impaired vascular function [1], and is associated with cardiovascular disease [2]. The retinal vasculature closely resembles the cerebral vasculature [3], and retinal vascular disease is associated with cerebrovascular disease [4].

The retinal vasculature plays a role in the pathogenesis of several eye diseases associated with OSA, including glaucoma, non-arteritic ischaemic optic neuropathy and diabetic retinopathy (DR) [5]. Endothelial dysfunction and impaired autoregulation are thought to underlie the development of DR [6]. Dynamic vessel analysis (DVA) allows assessment of retinal endothelial function using real-time recording of retinal vessel sizes in response to flickering light exposure [7]. DVA has demonstrated retinal endothelial dysfunction in patients with type 2 diabetes mellitus (T2DM) [8]. Whilst OSA has been shown to be a risk factor for more severe DR [9, 10], it is not known if this is due to OSA-induced retinal endothelial dysfunction.

Endothelial dysfunction is a precursor to atherosclerosis [11], along with retinal disease [6]. OSA causes endothelial dysfunction as measured using flow-mediated dilation (FMD) at the brachial artery [12, 13]. However, FMD is only weakly correlated with microvascular function, such as retinal vascular endothelial responses [14], which are important in the development of cardiovascular disease [15].

Retinal vasculature dysfunction may be important in the pathogenesis of eye diseases associated with OSA and provides a correlate of microvascular function important in cardiovascular disease. We aimed to investigate the effect of OSA on retinal vascular reactivity in patients without established eye disease or diabetes mellitus (DM).

## Methods

The retinal reactivity in OSA study was a multi-centre, double-blind, randomised, parallel controlled trial. It was prospectively registered (ISRCTN 78082983) and ethically approved (NHS REC 14/SC/1235). All participants provided written informed consent.

### Participants and screening

Participants were recruited from four hospital sleep clinics in the UK. Participants had an original diagnosis of moderate-to-severe OSA, had no known history of DM and had been treated with continuous positive airway pressure (CPAP) for more than 6 months with mean CPAP usage exceeding 4 h/night in the 30 days prior to screening. Participants underwent screening involving home overnight pulse oximetry to ensure that their OSA was well controlled on CPAP and to ensure return of OSA on stopping CPAP. Further details along with full inclusion and exclusion criteria are listed in the online supplement (Suppl. P3–4).

### Randomisation, intervention and blinding

Randomisations and interventions were performed as described in a previous CPAP withdrawal study [12]. Randomisation was carried out by an unblinded researcher not performing outcome assessments. Participants were randomised 1:1 using online randomisation software (http://www.sealedenvelope.com/) by minimised randomisation (minimised by the highest screening ODI off CPAP, < or ≥ 33/h; age, < or ≥ 60 years; BMI, < or ≥ 34 kg/m^2^). Participants were randomised at baseline to receive either continued therapeutic CPAP or sham CPAP. Further details of the intervention are included on the online supplement (Suppl. P4–5). Participants and the researcher performing outcome assessments were blind to treatment allocations. All participants were instructed to use their allocated replacement CPAP machine overnight for 14 days and their own non-trial CPAP machine was retained at the study site for the intervention period.

### Procedures

Full details of the study procedures and methodologies can be found in the online supplement (Suppl. P5–7).

#### Retinal vessel reactivity to flicker light stimulation

Dynamic vessel analysis (DVA, IMEDOS Systems, Jena, Germany) with a flickering light protocol [16] was performed during wakefulness at baseline and, at the same time of day, after 14 days of intervention. Full details of DVA analysis and flickering light protocol are provided in the online supplement. In brief, this enables continuous recordings of retinal vessel diameters before, during and after 20 s of flickering light, which is a powerful retinal metabolic stimulus which in health causes both retinal arteriolar and venular dilatation [16]. DVA assessment of retinal vessel diameters was also performed (before, during and after) whilst the patient’s hand was submerged for 1 min into ice cold water (0 °C) to elevate arterial blood pressure (see description of cold pressor test in the online supplement for full details).

#### Ocular and systemic patient characteristics

In addition to DVA, a detailed baseline and follow-up ophthalmic assessment was performed including visual fields assessment (HFA, Central 30–2 Threshold Test), intraocular pressure (rebound tonometry, I-CARE, Medline) and static retinal photography (50° retinal photography, full colour and red free, Zeiss FF450 + , Zeiss Meditech). Office blood pressure and heart rate were performed in triplicate after at least 5 min in a seated position. Participants were instructed to record home blood pressure and heart rate measurements in triplicate every morning in a seated position immediately after waking (Omron M10), from three mornings prior to their baseline visit until the end of follow-up. Participants were instructed to perform overnight pulse oximetry (300i; Konica Minolta) for each of the 14 intervention nights. Blood glucose measurements (random) were recorded at baseline and follow-up, and baseline glycosylated haemoglobin (HbA1c) levels were recorded. Baseline weight, height and neck circumference were recorded. Mean hours of pre-trial CPAP usage were recorded over the 30 days prior to randomisation.

### Outcomes

**Fig. 1:**
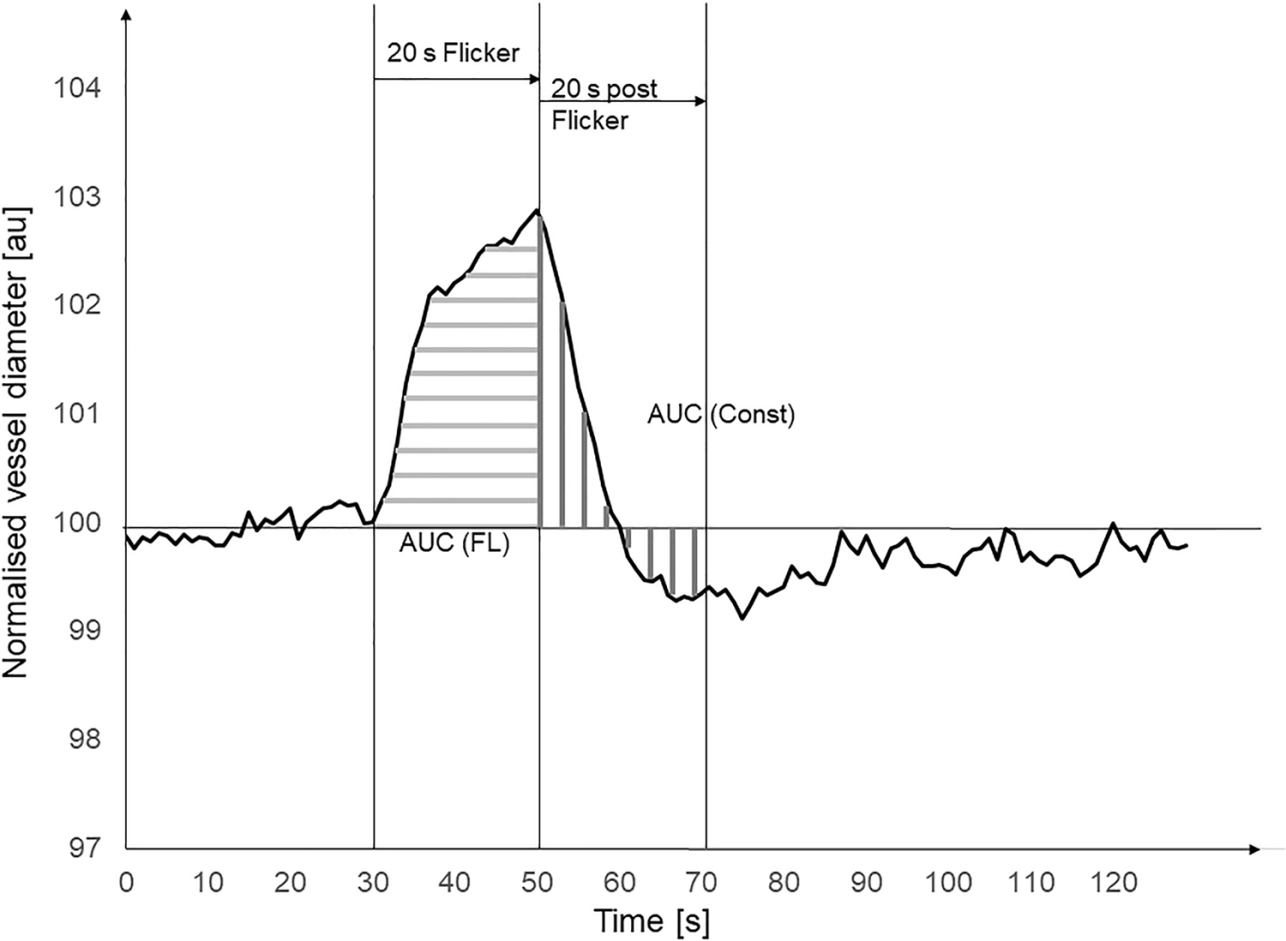
Schematic representation of the retinal vessel diameter and of the area under the curve measurements for primary and secondary outcome measures. AUC (FL), area under the curve for the 20 s during flickering light, which is the primary outcome. AUC (Const), area under the curve for the 20 s following flickering light

The primary outcome was the change from baseline to follow-up visit in retinal arteriolar diameter change to flicker light stimulation, defined as the area under the curve (AUC) during 20 s of the flickering light stimulation, averaged over three repeated flicker exposures, sham CPAP versus therapeutic CPAP (AUC (FL) see Fig. 1).

Secondary outcome measures included the AUC as defined as above for venular response to the flickering light protocol; the total arteriole AUC during and following the flickering light protocol; the total venular AUC during and following the flickering light protocol; and the area under the arterial curve following the end of the flicker light for a maximum of 20 s (AUC (Const) see Fig. 1). The maximal arteriolar dilation during the flickering light protocol, the maximal venular dilation during the flickering light protocol and the maximum arteriolar constriction following the flickering light all reported as the percentage change from baseline. The correlation of the primary outcome with the average overnight ODI during the last seven nights of CPAP withdrawal, the correlation of the primary outcome measure with the average overnight heart rate rises (rises > 6 bpm from baseline) and the correlation of the primary outcome measure with the average home morning blood pressure on the last three mornings of CPAP withdrawal, were assessed. Retinal arteriole and venous responses during and after a cold pressor test were exploratory outcomes.

### Statistics

Sample size estimation was based on data showing reduced retinal vascular reactivity of 1.4 ± 1.8% in participants with T2DM but without known retinopathy, compared to 3.2 ± 1.6% in controls [17]. In order not to miss a similar effect of OSA on the arteriole AUC during the flickering light protocol, with 90% power and with two-sided alpha of 0.05, 20 patients in each arm were required. Alternatively, brachial artery FMD is correlated to some extent with retinal reactivity [14], and CPAP withdrawal causes a 3.2% reduction in FMD from a baseline of 5.2 ± 2.6% [12]. In order not to miss a similar effect of OSA on the arteriole AUC during the flickering light protocol, with 90% power and with a two-sided alpha of 0.05, 15 patients in each arm were required. We therefore aimed to recruit over 40 participants, so to account for any dropout.

Statistical analysis was carried out using SPSS Version 26.0 (IBM, USA). Continuous data were assessed for normality and expressed as mean ± standard deviation where normally distributed and median (first quartile, third quartile) where non-normally distributed. Categorical data are expressed as number (percentage). Primary and secondary outcome analyses were performed using multi-variable linear regression adjusted with the dependent variable defined as the outcome variable at follow-up and the treatment effect modelled as the effect of sham CPAP versus therapeutic CPAP, with adjustments for baseline values, and further adjustments for OSA severity during screening, BMI, age and available relevant components of the Pocock cardiovascular risk score (smoking status, systolic blood pressure, prior cardiovascular disease) using an enter forward selection criteria (*p* < 0.10). A sensitivity analysis was performed excluding participants with HbA1c values indicative of new diagnoses of T2DM (HbA1c > 6.4 mmol/mol). Univariate correlations were assessed using Pearson’s or Spearman rank correlation as appropriate.

## Results

**Fig. 2:**
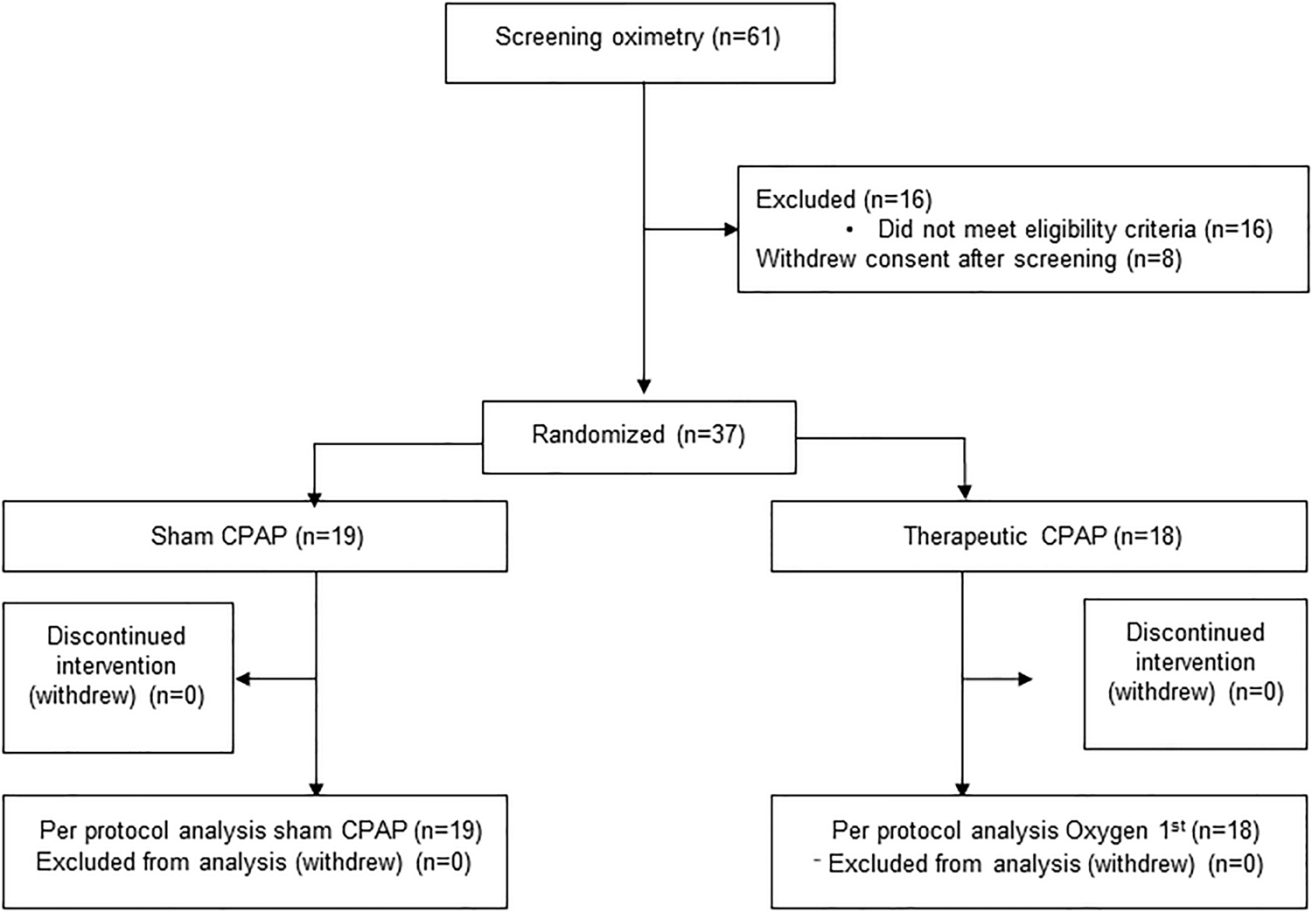
Study flow diagram showing potential participants who underwent screening oximetry. CPAP, continuous positive airway pressure

Thirty-seven participants were recruited from January 2015 until March 2020, when a decision was taken to close the study due to study-site closures resulting from the coronavirus pandemic. Details of the participants and randomisation are included in the study flow diagram (Fig. 2). Baseline characteristics were similar for both groups, with similar pre-trial OSA severity, OSA control and CPAP adherence (see Table 1).

**Table 1:** Baseline characteristics. Continuous data displayed as either mean ± standard deviation if normally distributed or median (first quartile, third quartile) if non-normally distributed. Categorical data displayed as number (percentage). *BMI*, body mass index; *CPAP*, continuous positive airway pressure; *IHD*, ischaemic heart disease; *HbA1c*, glycosylated haemoglobin; *ODI*, oxygen desaturation ≥ 4% index

	CPAP (*n* = 18)	Sham CPAP (*n* = 19)
Age (years)	61.3 ± 8.5	60.0 ± 8.8
Ethnicity		
- White	17 (94%)	18 (95%)
- Asian	1 (6%)	0 (0%)
- Hispanic	0 (0%)	1 (5%)
Gender		
- Male	14 (78%)	15 (79%)
- Female	4 (22%)	4 (21%)
Smoking status		
- Never smoker	9 (50%)	9 (47%)
- Ex-smoker	9 (50%)	8 (42%)
- Current smoker	0 (0%)	2 (11%)
Smoking (pack years)	18.0 (9.3, 28.5)	17.0 (8.8, 27.5)
Hypertension	11 (61%)	9 (47%)
Hypercholesterolemia	2 (11%)	1 (5%)
IHD or stroke	2 (11%)	0 (0%)
Atrial fibrillation	1 (6%)	0 (0%)
Neck circumference (cm)	43.1 ± 4.9	43.4 ± 3.6
BMI (kg/m^2^)	35.8 ± 4.9	36.6 ± 5.2
HbA1C (%)	5.5 (5.3, 5.8)	5.7 (5.2, 5.9)
Random glucose measurement (mg/dL)	102.6 (84.6, 119.7)	111.6 (93.6, 131.4)
CPAP compliance (h/n)	6.4 ± 1.1	6.1 ± 1.4
Screening ODI off CPAP (/h)	32.7 (22.9, 47.7)	37.5 (27.8, 70.3)
Screening ODI on CPAP (/h)	5.1 ± 2.4	5.1 ± 2.3
Baseline ESS	5.4 ± 4.5	5.7 ± 4.2

### Baseline ocular assessments

Baseline intraocular pressure and visual field measurements were conducted to exclude significant baseline eye disease. Intraocular pressure and visual fields were comparable at baseline between both groups and within the normal ranges (Table 2).

**Table 2:** Baseline intraocular pressure and visual field assessments. Data expressed as either mean ± standard deviation if normally distributed or median (first quartile, third quartile) if not normally distributed, and categorical data displayed as number (percentage). *MD*, mean deviation; *PSD*, pattern standard deviation

	CPAP	Sham CPAP
*Intraocular pressure*		
Right intraocular pressure (mmHg)	16.7 ± 2.8	15.8 ± 3.7
Left intraocular pressure (mmHg)	16.2 ± 3.0	15.5 ± 4.3
*Visual field*		
Foveal threshold (dB)	32.8 ± 2.5	33.3 ± 3.3
30–2 MD (dB)	− 1.6 ± 1.8	− 1.7 ± 1.4
30–2 PSD (dB)	2.0 (1.6, 2.3)	2.0 (1.7, 2.5)

### Primary and secondary outcome measures

One patient in the sham CPAP group and two patients in the CPAP group had technically insufficient traces that did not allow measurement of arterial responses to flickering light, giving a total of 34 patients in whom primary outcome assessment was possible.

At baseline, there was a chance difference in the AUC for the mean arteriolar response to the flickering light protocol for sham CPAP (28.2 ± 26.2 arbitrary units or AU) compared to therapeutic CPAP (50.7 ± 30.3 AU).

**Fig. 3:**
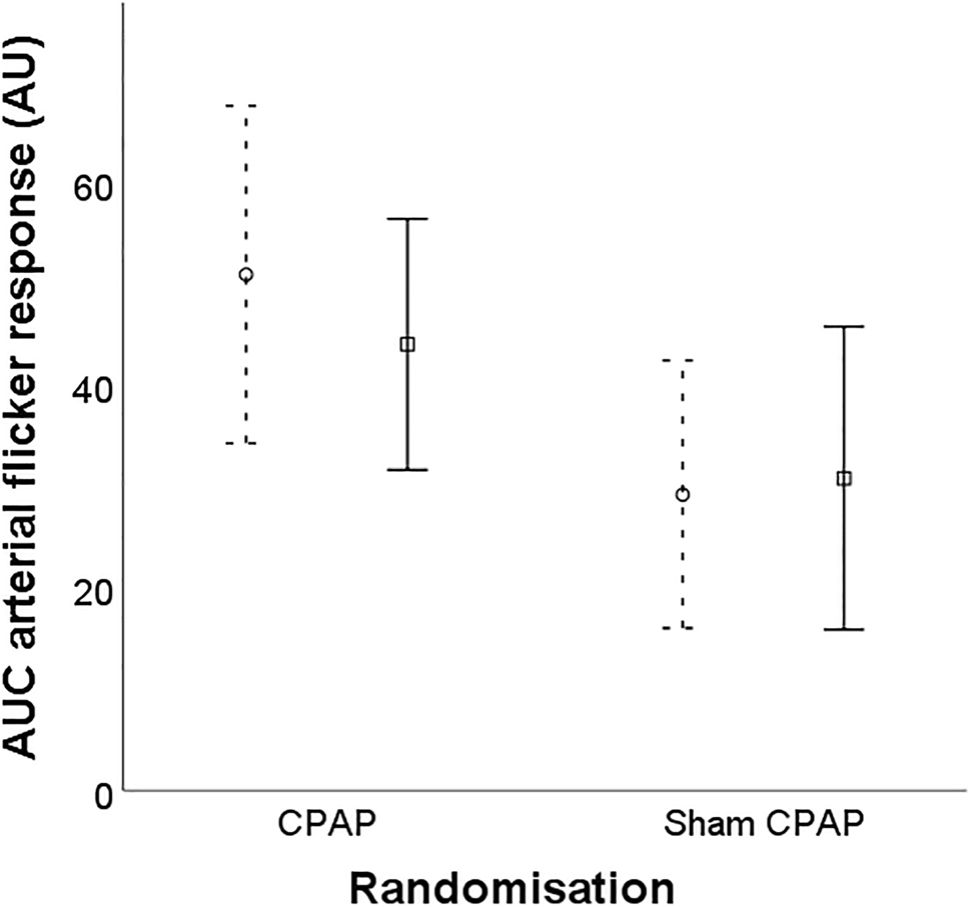
Plot showing the area under the curve value increase in arteriole diameter during flickering exposure, proportional to baseline diameter, for CPAP and sham CPAP. Circles and squares represent baseline and follow-up mean values respectively with dotted and solid lines representing baseline and follow-up 95% confidence intervals, respectively. AUC, area under the curve; CPAP, continuous positive airway pressure

There was no significant change in the primary outcome with no effect of sham CPAP on the change in the AUC for arteriolar response to flickering light of + 3.8 AU (95% CI − 10.6 to + 18.2, *p* = 0.59, Fig. 3) versus continued CPAP.

There were no significant differences in the treatment effect of sham CPAP in other secondary outcome measures (see Table 3).

**Table 3:** Primary and secondary outcome data showing baseline and follow-up values for optic assessments along with the modelled treatment effect and 95% CI. Data displayed as either mean ± standard deviation if normally distributed or median (first quartile, third quartile) if non-normally distributed. Treatment effect of CPAP withdrawal modelled using multi-variable linear regression with follow-up value as the dependent variable, treatment (CPAP or sham) as a fixed effect, with adjustment for baseline value and further adjustments for age, gender, stroke, IHD, HbA1c, smoking status, systolic blood pressure, BMI and OSA severity during screening using an enter forward selection criteria (*p* < 0.10). *AUC*, area under the curve; *AVR*, central retinal artery to central retinal vein ratio; *CPAP*, continuous positive airway pressure; *CRAE*, central retinal artery equivalent; *CRVE*, central retinal vein equivalent

	CPAP		Sham CPAP		Treatment effect	95% CI	*p* value
	Baseline	Follow-up	Baseline	Follow-up			
*Primary outcome: dynamic retinal arteriole response to flickering light protocol*							
AUC Flicker arterial	50.7 ± 30.3	44.0 ± 23.2	28.2 ± 26.2	30.8 ± 30.1	+ 3.8	− 10.6 to + 18.2	0.59
*Secondary outcomes: dynamic retinal arteriole response to flickering light protocol*							
Maximal dilation (%)	4.4 ± 1.7	4.3 ± 1.3	3.0 ± 1.5	3.2 ± 2.2	− 0.1	− 1.3 to + 1.1	0.87
AUC Flicker constriction	− 4.8 (− 13.7, + 3.8)	− 1.2 (− 18.1, + 10.3)	− 15.8 (− 34.4, − 5.5)	− 11.3 (− 35.4, − 5.8)	− 5.9	− 16.4 to + 4.5	0.25
Maximal constriction (%)	− 1.0 (− 1.7, − 0.7)	− 1.0 (− 2.2, − 0.0)	− 1.7 (− 3.0, − 1.2)	− 2.3 (− 3.3, − 1.1)	− 0.6	− 1.1 to + 0.0	0.05
Baseline diameter fluctuation	2.5 ± 1.4	2.4 ± 1.1	2.0 ± 1.1	2.3 ± 1.1	+ 0.0	− 0.6 to + 0.7	0.95
Dilation amplitude	5.2 (4.3, 7.4)	5.0 (3.5, 7.3)	4.4 (3.8, 6.3)	5.0 (3.5, 6.7)	+ 0.6	− 0.8 to + 2.0	0.38
Arteriole size	109.0 ± 18.3	109.5 ± 15.7	107.6 ± 13.5	108.2 ± 13.9	− 1.3	− 5.4 to + 2.8	0.53
*Secondary outcomes: dynamic retinal venule response to flickering light protocol*							
AUC Flicker	51.7 ± 23.1	49.3 ± 25.2	43.2 ± 18.9	36.8 ± 18.1	− 6.0	− 20.1 to + 8.0	0.39
Max dilation (%)	4.7 ± 1.6	4.9 ± 2.0	4.4 ± 1.9	4.1 ± 1.5	− 0.7	− 1.9 to + 0.6	0.28
Baseline diameter fluctuation	1.4 (1.0, 2.2)	1.8 (1.3, 2.2)	1.5 (1.2, 2.4)	1.8 (1.3, 3.6)	− 0.1	− 0.9 to + 0.6	0.71
Venule size	139.7 ± 23.8	143.1 ± 24.3	146.7 ± 19.0	147.4 ± 20.1	− 1.3	− 4.3 to 1.6	0.37
*Static retinal photography*							
CRAE	150.0 (145.5, 163.5)	152.5 (142.3, 169.0)	160.0 (154.0, 166.0)	156.0 (151.0, 169.0)	− 2.3	− 7.0 to + 2.4	0.33
CRVE	203.7 ± 20.7	206.0 ± 23.1	211.1 ± 17.0	211.2 ± 18.1	+ 0.8	− 3.3 to + 5.0	0.68
AVR	0.75 (0.72, 0.78)	0.77 (0.72, 0.79)	0.76 (0.72, 0.79)	0.75 (0.71, 0.78)	− 0.01	− 0.03, + 0.01	0.22

### CPAP withdrawal

Full results of the effect of CPAP withdrawal on overnight pulse oximetry, Epworth sleepiness score (ESS), home morning blood pressure and heart rate measurements and office blood pressure and heart rate measurements are shown in Table S1 in the online supplement.

CPAP withdrawal led to a marked return of OSA and an increase in daytime sleepiness, with an increase in the ODI of 27.5/h (95% CI 16.7 to 38.3, *p* < 0.001) and an increase in the ESS of 3.5 points (95% CI 1.7 to 5.4, *p* = 0.001).

CPAP withdrawal significantly increased home morning heart rate and office heart rate (*p* = 0.01 and 0.02, respectively), but the rises in home or office systolic or diastolic blood pressure did not reach statistical significance (see Table S1 in the online supplement).

There were no significant correlations between the change in the AUC of the arteriole response to the flicker light protocol and the trial ODI, the heart rate rises (> 6 pm) index (a marker of ‘autonomic’ arousals during sleep), the change in home morning systolic blood pressure or the change in home morning diastolic blood pressure in the sham CPAP arm (Table S2 in the online supplement).

### Cold pressor test

As expected, the cold pressor test statistically significantly increased systolic and diastolic blood pressure (Table S3 in the online supplement*).* Retinal arteriole and venous vessel diameters were comparable at baseline during 1 min of recording prior to cold pressor test at both the baseline and follow-up visits (Table S4 in the online supplement)*.* CPAP withdrawal caused an unexpected small but significant increase in the maximal retinal arteriole vasoconstriction during the cold pressor test (treatment effect 1.3%, 95% CI 0.2 to 2.3%, *p* = 0.02). There were no other significant effects of CPAP withdrawal on cold pressor arteriole or venous responses (Table S4 in the online supplement).

### Baseline glycaemic assessment

HbA1C measurements were taken at baseline to identify those at risk of undiagnosed diabetes. HbA1C measurements were available for 36 of the 37 participants. In one participant randomised to sham CPAP, the HbA1c sample was inadequate. Two participants (one randomised to sham CPAP with a value of 7.4% and one randomised to therapeutic CPAP with a value of 6.6%) had HbA1c values above 6.4%, consistent with undiagnosed T2DM.

A sensitivity analysis excluding the two participants with HbA1c values > 6.4% was conducted for the primary outcome measure. This showed similar results, with no significant change in AUC for the arteriolar response to flickering light with a treatment effect of sham CPAP of 5.5 AU (95% CI − 10.0 to + 21.0, *p* = 0.47).

## Discussion

Fourteen days of CPAP withdrawal had no significant effect on retinal microvascular function measured with the flickering light protocol. CPAP withdrawal caused a clear return of intermittent hypoxia, daytime sleepiness and a rise in morning heart rate consistent with returning moderate-to-severe OSA, both in terms of symptoms and physiological effect. Despite these effects of CPAP withdrawal, it had no significant effect on the primary outcome or any other measure of retinal microvascular response to flickering light; furthermore, it did not reduce retinal arteriolar vasoconstriction in response to the cold pressor test, and in fact increased it. To our knowledge, this is the first randomised controlled trial to report on the effects of stopping CPAP treatment for 14 days, in patients with OSA on both static and dynamic retinal vessel parameters.

### Ocular endothelial function

Retinal vessel responses to flickering light are a measure of microvascular nitric oxide–mediated endothelial function [18]. A small number of case-cohort studies exploring choroidal and central retinal blood flow using laser colour Doppler or applanation tonometry in OSA have been summarised previously [19], but these do not directly assess retinal microvascular responses. Optical coherence tomography angiography (OCTA) has also been used to assess the retinal microvasculature in a small number of non-randomised studies, but these are limited by matching [20, 21], or by characterising patients only by OSA risk rather than sleep studies [22]. Retrobulbar blood flow velocities have been reported to be increased in moderate-to-severe OSA in comparison with unmatched controls of similar age [23].

Fourteen days of exposure to intermittent hypoxia in rats showed oxidative stress in the ophthalmic artery associated with endothelial dysfunction mediated through nitric oxide and endothelium-derived hyperpolarising factor pathways [24]. However, the role of oxidative stress in OSA-mediated ocular vascular disease is contentious [25, 26], and such animal models often induce more marked intermittent hypoxia than seen in human OSA, and do not model for the correct associated rises in carbon dioxide levels that would generate vasodilation [27]. In addition, there are substantial differences in the blood supply distribution to the retina in rats compared with humans [28].

None of these experiments adequately examine the effects of human OSA on the retinal microvasculature. OSA has been reported to decrease the static arterio-venous ratio and attenuating retinal vascular pulsation [29]. Furthermore, patients with severe OSA who were not treated with CPAP had progressive reductions in static arterio-venous ratio, compared with those on CPAP, but CPAP had no effect on retinal vessel pulsatility [30]. There are key differences between these studies and ours. First, the measured arterio-venous ratio is not a dynamic measurement of retinal vascular function, unlike our dynamic retinal vessel analyses. Retinal pulsatility may be a measure of retinal vascular function but approaches to measure pulsatility are not standardised and are dependent on vessel diameter, location and axial length. Finally, these previous reports were not randomised as our study was. Comparing the effect of CPAP in non-randomised patients who are either on or not on CPAP is prone to bias and other factors. Nevertheless, these studies highlight the possibility that OSA may have deleterious effects on the retinal vasculature which were not apparent after 14 days of CPAP withdrawal.

### Comparisons with systemic endothelial function

OSA impairs endothelial function as measured by FMD in the brachial artery [31, 32]. For example, in contrast to our findings, an almost identical experimental paradigm found a 61% reduction in FMD after 14 days of CPAP withdrawal [12].

CPAP withdrawal, as a model of returning OSA, appears to have contrasting effects on endothelial function. There are marked reductions in macrovascular endothelial function as measured by brachial artery FMD [12], but no effect on several measures of microvascular function including cerebral vascular reactivity [33], myocardial perfusion and renal and dermal microvascular function [34], and no significant effect on retinal microvascular function in our study. These contrasting findings support the concept of differing effects of OSA on endothelial function in microvascular and macrovascular vessels.

Differing contributions of nitric oxide in macrovascular and microvascular endothelial function could explain these differences. Nitric oxide blockade abolishes macrovascular endothelial responses [35, 36], whilst only partially attenuating retinal [18], and other microvascular endothelial responses [37]. In addition, glial cells—which are not present outside of the nervous system—play an important role in retinal hyperaemia [38], which may contribute to differing responses between retinal arterioles and the brachial artery.

### Retinal pressure autoregulation

Retinal blood flow autoregulation has both metabolic—as assessed by the flickering light protocol—and blood pressure components. Pressure autoregulation refers to the maintenance of a constant blood flow despite varied ocular perfusion pressures. Retinal vessel vasoconstriction in response to increased central blood pressure is impaired in retinal vascular disease [39]. However, our study showed that CPAP withdrawal did not impair cold pressor–induced vasoconstriction. Retinal vasoconstriction in response to raised central blood pressure diminishes with age [40], and it is possible that the older age of our participants accounts for the small vasoconstriction responses we observed in response to cold pressor testing. Intriguingly, we observed a paradoxical increase in vasoconstriction with CPAP withdrawal. This was an exploratory outcome and requires validation. This suggests that OSA does not impair retinal pressure autoregulation, at least in the short term and in the absence of eye disease.

### Diabetic retinopathy (DR) and OSA

OSA is associated with increased risk of more advanced DR [9], and may be associated with an increased risk of DR [9, 41]. The exact mechanisms for accelerated retinopathy are not clear but might include endothelial dysfunction [5]. Both DR [42, 43] and probably DM alone [17] are associated with decreased retinal vascular reactivity, as a marker of endothelial function. A key aim of this study was to understand if OSA impairs retinal vascular reactivity in patients free from T2DM. Our data shows that short-term return of OSA during 14 days of CPAP withdrawal does not alter static retinal vessel calibres or retinal vessel reactivity to flicker light. However, there may be different effects on these parameters in those with underlying diabetes mellitus, or those exposed to OSA in the longer term. The only randomised trial to examine the longer term effects of CPAP treatment for OSA on progression of DR showed no benefit; however, it was limited by CPAP usage of 3.2 h/night [44], meaning that further work with long-term follow-up and optimised CPAP adherence is needed.

### Strengths and limitations of our study

This study has strengths and limitations. The use of a robust randomised design, the inclusion of patients with excellent CPAP compliance and the detailed and standardised measurements of pulse oximetry tracings and ophthalmic parameters are strengths of this study [45]. Our study was limited by a small sample size and early closure, three patients short of its recruitment target. However, our target allowed from drop-outs, of which there were none and it was powered to show an OSA-related reduction in retinal reactivity of similar magnitude to the effect of DM [17], and to show a similar magnitude of reduction in endothelial function as previously reported in the brachial artery during CPAP withdrawal [12]. There are no prior data solely exploring the effects of OSA on retinal vascular reactivity and the minimal clinically important change in retinal vascular response to the flickering light protocol is not known. The 95% CI for change in arteriolar AUC in response to flickering light in the sham CPAP arm was − 10.6 to + 18.2, which represents up to a possible 37.6% deterioration relative to the baseline value. Therefore, we are confident that we have excluded a reduction in retinal vascular reactivity during CPAP withdrawal of a similar magnitude to the 61.4% decline in endothelial function measured in a similar experimental paradigm using flow-mediated dilation at the brachial artery [12]. This supports the view that the effect of OSA on endothelial function varies by vascular bed. In our study, CPAP was only withdrawn for 14 days, which is a short duration compared to the long-term effects of T2DM on retinal vasculature. It may be that 14 days of CPAP withdrawal do not replicate the longer term effects of untreated OSA; however, there are ethical concerns about withdrawing CPAP for longer periods of time. In the future, in order to gain further insights into the effects of OSA on the retinal vasculature, it may be necessary to utilise new approaches and techniques. Propensity matching has been suggested as an alternate method to traditional RCTs [46], but this is controversial [47]. Techniques such as ocular coherence tomography angiography have the potential to assist with determining the effects of OSA on the retinal vasculature and are becoming more readily available.

## Conclusions

Fourteen nights of CPAP withdrawal had no significant effect on retinal microvascular endothelial function in response to a flickering light protocol. CPAP withdrawal did cause a marked increase in intermittent hypoxia, daytime sleepiness and heart rate, consistent with the return of OSA and its consequences. These results contrast with the finding that CPAP withdrawal causes endothelial dysfunction at the brachial artery. This suggests that OSA may have different effects on endothelial function in the microvasculature, as compared to larger conduit vessels, and further work is needed to confirm this.

## Supplementary Information

Below is the link to the electronic supplementary material.

Supplementary file1 (PDF 317 KB)
