# Supplementary material for: Effect of obstructive sleep apnoea on retinal microvascular function: a randomised controlled trial
Source: Graefes Arch Clin Exp Ophthalmol. 2022 Feb 24;260(7):2129–39. doi: 10.1007/s00417-022-05596-8 (PMC8866916; doi:10.1007/s00417-022-05596-8)
Supplement: Supplementary file 1 — Supplementary file1 (PDF 317 KB) [file 417_2022_5596_MOESM1_ESM.pdf]

Effect of obstructive sleep apnoea on retinal microvascular function: a randomised controlled trial

Chris D Turnbull[1,2], James A Stockley[3], Shyam Madathil[3], Syed SA Huq[3], Brendan G Cooper[3], Asad Ali[4], Simon Wharton[5], John R Stradling[2], Rebekka Heitmar[6]

1. Nuffield Department of Medicine, University of Oxford, Oxford, United Kingdom
2. NIHR Biomedical Research Centre University of Oxford, Oxford, United Kingdom
3. Lung Function & Sleep, Queen Elizabeth Hospital, University Hospitals Birmingham NHSFT, Birmingham, B15 2GW, West Midlands, United Kingdom.
4. Department of Sleep and Respiratory Medicine, University Hospital Coventry and Warwickshire, Coventry, CV2 2DX, Warwickshire, United Kingdom.
5. Sleep Department, Heartlands Hospital, University Hospitals Birmingham NHSFT, Birmingham, B15 2GW, West Midlands, United Kingdom
6. University of Huddersfield, School of Applied Sciences, Department of Optometry and Vision Sciences, Huddersfield, United Kingdom

Corresponding author: Chris D Turnbull christopher.turnbull@ouh.nhs.uk

https://orcid.org/0000-0001-8942-5424

Contents

Table of Contents

Methods*.. *3

Participants and screening 3

Inclusion and exclusion criteria 3

Inclusion Criteria 3

Exclusion Criteria.. 4

Randomisation, Intervention and Blinding 4

Procedures 5

Retinal Vessel Analyses 5

Cold-pressor 6

Visual fields assessment.. 6

Intraocular pressure 6

Static retinal photography 7

Table E1: Baseline, follow-up and the effects of CPAP withdrawal on overnight oximetry, Epworth sleepiness score, home morning blood pressure and heart rate measurements, and office blood pressure and heart rate measurements. **8**

Table E2: The Spearman's rank correlations between the change in the area under the curve of the arteriole flicker response from baseline to follow-up with the ODI, overnight heart rate rises (>6bpm), and the change in home blood pressure. **8**

Table E3: The effect of cold pressor on systolic and diastolic blood pressure at baseline and follow-up visits in the sham and CPAP arms. **9**

Table E4: Retinal arteriole and venous vessel diameters before, during and after cold-pressor test at baseline and follow-up visits in the CPAP and sham CPAP groups. **10**

Methods

Participants and screening

In original protocol, participants were required to have had an original diagnosis of moderate-to-severe obstructive sleep apnoea (OSA), no known history of diabetes mellitus (DM), and had been treated with continuous positive airway pressure (CPAP) for more than 12 months with mean CPAP usage exceeding 4 h/night in the 30 days prior to screening. In February 2016 the protocol was amended to allow inclusion of patients after 6 months or more of CPAP therapy to increase recruitment. Participants completed one week of home overnight screening pulse oximetry (300i; Konica Minolta). This consisted of three nights oximetry whilst receiving CPAP, followed by four nights without CPAP. Eligible participants had an oxygen desaturation index ≥4% (ODI) less than 10 /h on all three nights on CPAP and an ODI exceeding 20 /h on at least one of four nights off CPAP.

Inclusion and exclusion criteria

Full inclusion and exclusion criteria are listed below.

Inclusion Criteria

1. Objectively confirmed obstructive sleep apnoea (at the time of original diagnosis) with an oxygen desaturation index (ODI, >4% dips) or AHI of >20 (this threshold will exclude participants with borderline OSA, in whom there may be little experimental effect)

2. Currently >20/h oxygen desaturations (>4% dips) returning on any night during home nocturnal pulse oximetry performed for a 4-night period without CPAP, prior to entry into the study.

3. Treated with CPAP for more than 6 months, minimum compliance 4h per night.

4. ODI <10 during treatment (obtained during the preliminary week of oximetry monitoring, from the first 3 nights of oximetry monitoring while on CPAP, before the 4 nights CPAP withdrawal).

5. Age between 20 and 75 years at trial entry.

6. Written informed consent.

Exclusion Criteria

1. Previous ventilatory failure (awake resting arterial oxygen saturation <93% or arterial PCO2> 6kPa) or severe respiratory disorders other than OSA.

2. Unstable, untreated coronary or peripheral artery disease, severe arterial hypertension (>180/110mmHg), severe arterial hypotension (<90/60mmHg).

3. Previously diagnosed with Cheyne-Stokes breathing.

4. Current professional driver

5. History of any sleep-related driving accident or other accident.

6. Acute inflammatory disease

7. Acute or chronic hepatic or renal disease.

8. Known type 1 or 2 diabetes (likely to have low retinal reactivity even on CPAP)

9. Known severe vascular disease (likely to have low retinal reactivity even on CPAP)

10. Mental or physical disability precluding informed consent or compliance with the protocol.

11. Non-feasible trial follow-up (for example, distance from follow-up centre, physical inability).

12. Epilepsy: flickering light used for retinal provocation could in theory lead to a seizure.

13. Lens opacities: this could lead to insufficient contrast and make it impossible to image the retinal vasculature.

Randomisation, Intervention and Blinding

Patients randomised to continued therapeutic CPAP were issued a replacement CPAP machine which delivered auto-adjusting positive airway pressure, with pressures ranging from 4 to 20 cmH_2O

(ResMed Autoset S9, Abingdon, UK). Patients randomised to sham CPAP were issued a replacement sham CPAP machine. Sham CPAP was delivered by an identical device to the continued CPAP group, but set to 4cmH_2O with additional holes (by the mask) lowering the effective pressure delivered to ≤ 1cmH_2O (ResMed Autoset S9, Abingdon, UK). This has been shown to be an effective method of participant blinding in previous similar experiments [1].

Procedures

Retinal Vessel Analyses

Retinal vessel diameters were measured continuously at a sampling rate of 25 Hz (IMEDOS Systems, Jena, Germany). Stimulation of retinal blood vessels was done by optoelectronic interruption of the green fundus illumination used by the retinal vessel analyser (RVA) resulting in a flicker light (FL) provocation with a 12.5 Hz frequency [2-4]. Following image focussing one vessel segment of each, a retinal artery and vein was chosen at a distance of 1-2 disc diameters away from the margins of the optic nerve head. Baseline diameters of both the artery and vein were recorded according to the standard RVA protocol [4], for 50 seconds and then followed by 3 cycles of 20 second flicker provocation with each 80 seconds recovery time. Resulting in a 350 second measuring period during which the fellow eye was occluded to improve patient fixation. From these diameter recordings of arteries and veins the values for baseline diameter fluctuation (BDF) maximum dilation (MD), maximum constriction (MC) and dilation amplitude (DA), were calculated for averaged flicker responses [5].

Areas under the curve parameters of retinal arteriolar responses to FL were calculated using the averaged time course of all three FL cycles: AUC(FL): Area under the curve of the averaged profile during 20 second flicker, AUC(Constriction): Area under the curve of 20 second duration following flicker cessation (example see *Figure 1*).

Cold-pressor

To assess retinal vessel diameters with a modified cold pressor test we continuously recorded retinal arteries and veins under constant illumination with a red-free filter in place (IMEDOS Systems, Jena, Germany). The total recording length was set to 300 seconds where the first 60 seconds constituted the baseline, with the following 60 seconds of recording conducted whilst the patient's hand was submerged into ice cold water (0° Celsius), with the final 180 seconds of recording acting as the recovery period. The baseline values were calculated by averaging the last 30 seconds of the first 60 seconds of the baseline. The percentage changes compared to baseline, were calculated by averaging the first 30 seconds of ice water exposure and the first 30 seconds post ice water exposure.

Visual fields assessment

We carried out a number of procedures to assess for any ophthalmological abnormalities that might have invalidated our primary outcome assessment. These included visual field assessment, intraocular pressure measurements, and static retinal photography as detailed below.

Visual field data was obtained using the HFA2 (Zeiss Meditech) with the Central 30-2 Threshold Test protocol. All patients had previously undergone visual field testing (i.e. were not visual field naïve). In addition, we obtained a macula threshold on each visit prior to the visual field test starting. The following visual field data quality indices were required for results to be included in the statistical analyses: test results with poor reliability criteria defined as false positives (FPs) or false negatives (FNs) >20% and fixation losses (FLs) >30% were excluded from data analysis.

Intraocular pressure

Intra ocular pressure was measured using a rebound tonometer (I-CARE, Medline, UK). Six consecutive measurements per eye were obtained for each patient in a sitting position.

Static retinal photography

Retinal photographs were obtained using a Zeiss FF450+ fundus camera (Zeiss Meditech) following full pupil dilation. For the purpose of retinal vessel analysis, we obtained monochromatic (red-free) images with the camera angle set at 50 degree and the optic nerve head (ONH) centred. Summarised retinal vessel calibres of retinal arteries (central retinal artery equivalent=CRAE), veins (central retinal vein equivalent=CRVE) and the retinal artery-to-vein ratio (AVR) were calculated according to a standard protocol [6]. In brief, we included only the six largest arteries (to calculate CRAE) and six largest veins (to calculate CRVE) to calculate retinal vessel indices using a semi-automated software (VesselMap, IMEDOS SYSTEMS, Jena, Germany) and their inbuilt repeat measurement tool (so to select the same vessels for each visit).

Results

	CPAP		Sham CPAP		Treatment effect	95%CI	p value
	Baseline	Follow-up	Baseline	Follow-up			
Overnight pulse oximetry values							
ODI (/h)	-	5.1 (3.1, 8.0)	-	22.8 (16.4, 47.1)	+27.5	+16.7 to +38.3	<0.001
Mean SaO_2 (%)	-	94.9±1.6	-	92.9±2.1	-1.8	-3.1 to -0.6	0.006
Time SaO_2<90% (%)	-	1.0 (0.2, 3.0)	-	10.6 (3.7, 20.4)	+11.7	+3.9 to +19.5	0.004
Heart rate rises >6pbm (/h)	-	18.3±10.0	-	36.0±19.5	+19.1	+8.8 to +29.4	0.001
Epworth Sleepiness Score							
ESS	5.4±4.5	5.2±4.0	5.7±4.2	8.7±6.1	+3.5	+1.7 to +5.4	0.001
Home morning blood pressure and heart rate recordings							
Home systolic BP (mmHg)	137.6±16.6	138.0±14.3	125.3±13.1	131.2±15.5	+5.0	-1.0 to +11.0	0.10
Home diastolic BP (mmHg)	81.0±7.7	82.5±7.4	78.3±8.1	83.1±8.8	+2.8	-2.0 to +7.5	0.24

Home heart rate (bpm)	63.1±7.8	64.5±10.1	67.9±9.0	74.2±10.5	+6.4	+1.5 to +11.4	0.01
Office blood pressure and heart rate recordings							
Office systolic BP (mmHg)	132.9±17.8	131.7±18.8	129.2±13.5	127.9±15.8	-0.9	-9.0 to +7.3	0.83
Office diastolic BP (mmHg)	76.8±8.4	75.1±9.4	79.1±8.8	80.8±10.0	+3.6	-0.8 to +8.0	0.11
Office heart rate (bpm)	63.3±10.2	62.3±8.8	70.3±12.2	73.4±10.9	+6.5	+1.2 to +11.8	0.02

Table E1: Baseline, follow-up and the effects of CPAP withdrawal on overnight oximetry, Epworth sleepiness score, home morning blood pressure and heart rate measurements, and office blood pressure and heart rate measurements. Data expressed as either mean ± standard deviation if normally distributed or median (first quartile, third quartile) if not normally distributed, and categorical data displayed as number (percentage). Treatment effect of CPAP withdrawal modelled using multivariable linear regression with follow-up value as the dependent variable, treatment (CPAP or sham) as a fixed effect and adjustment for baseline value if applicable.

	Mean ODI (/h)	Mean heart rate rises (/h)	Change home systolic BP (mmHg)	Change in diastolic BP (mmHg)
Change in the area under curve of the arteriole flicker response	rho=-0.22 p=0.38	rho=-0.36 p=0.14	rho=-0.28 p=0.28	rho=-0.16 p=0.55

Table E2: The Spearman's rank correlations between the change in the area under the curve of the arteriole flicker response from baseline to follow-up with the ODI, overnight heart rate rises (>6bpm), and the change in home blood pressure. Correlations were assessed in the sham CPAP group only. ODI=oxygen desaturation index ≥4%.

	Blood pressure before and during cold pressor				
	Beginning	Maximum	Mean increase	95% CI	P
Systolic blood pressure (mmHg)					
CPAP at baseline visit	143.5±19.2	150.3±19.9	+6.8	+3.7 to +9.9	<0.001
CPAP at follow-up visit	145.6±23.9	148.2±19.8	+2.7	-6.7 to +12.0	0.56
Sham at baseline visit	140.2±16.6	145.8±16.8	+5.6	+2.0 to +9.2	0.005
Sham at follow-up visit	135.8±14.3	146.1±16.5	+10.3	+6.8 to +13.7	<0.001
Diastolic blood pressure (mmHg)					
CPAP at baseline visit	85.0±9.4	89.8±10.1	+4.8	+2.4 to +7.1	0.001
CPAP at follow-up visit	89.2±21.4	86.1±9.0	-3.1	-14.2 to +8.0	0.56
Sham at baseline visit	87.1±10.5	90.2±9.5	+3.1	+0.2 to +6.0	0.04
Sham at follow-up visit	88.0±10.0	93.5±9.1	+5.6	+2.5 to +8.6	0.001

Table E3: The effect of cold pressor on systolic and diastolic blood pressure at baseline and follow-up visits in the sham and CPAP arms. Data compared using paired t-tests. Data are expressed as mean ± standard deviation, or mean and 95%CI. CPAP= continuous positive airway pressure, 95%CI = 95% confidence interval.

	CPAP		Sham CPAP		Treatment effect	95%CI	p value
	Baseline	Follow-up	Baseline	Follow-up			
Vessel diameter before cold-pressor test							
Arteriole (AU)	109.1 ±17.2	109.1 ±16.9	105.9 ±12.9	106.4 ±10.4			
Venous (AU)	141.8 ±23.9	141.1 ±24.3	147.8 ±19.9	146.6 ±18.1			
Change in vessel diameter during cold-pressor test							
Arteriole (%)	-0.9±1.4	-0.3±1.6	-0.8±2.3	-1.5±1.4	-1.3	-2.3 to -0.2	0.02
Venous (%)	-1.0 (-1.0, +0.3)	0.0 (-1.0, 0.0)	-1.0 (-1.0, 0.0)	-1.0 (-1.0, 0.0)	-0.3	-0.9 to +0.3	0.39
Change in vessel diameter after cold-pressor test							
Arteriole (%)	-1.0 (-1.0, 0.0)	-0.5 (-1.0, 0.0)	0.0 (-1.0, +0.3)	-1.0 (-1.3, +0.3)	-0.1	-1.4 to +1.2	0.89
Venous (%)	-1.0 (-2.0, 0.0)	-1.0 (-1.3, 0.0)	-1.0 (-1.0, +0.0)	-1.0 (-1.0, 0.0)	-0.6	-1.5 to +0.3	0.18

Table E4: Retinal arteriole and venous vessel diameters before, during and after cold-pressor test at baseline and follow-up visits in the CPAP and sham CPAP groups. Diameters during and after cold pressor test are reported as % change from baseline and the treatment effect is modelled using multi-variable linear regression adjusted as described for the primary outcome in statistical analysis section of the methods. Data are displayed as mean ± standard deviation or median (first quartile, third quartile). CPAP=continuous positive airway pressure, 95%CI=95% confidence interval.

References

1. Gaisl T, Rejmer P, Thiel S, Haile SR, Osswald M, Roos M, Bloch KE, Stradling JR, Kohler M. Effects of suboptimal adherence of CPAP therapy on symptoms of obstructive sleep apnoea: a randomised, double-blind, controlled trial. Eur Respir J. 2020;55(3).
2. Blum M, Bachmann K, Wintzer D, Riemer T, Vilser W, Strobel J. Noninvasive measurement of the Bayliss effect in retinal autoregulation. Graefes Arch Clin Exp Ophthalmol. 1999;237(4):296-300.
3. Seifertl BU, Vilser W. Retinal Vessel Analyzer (RVA)--design and function. Biomed Tech (Berl). 2002;47 Suppl 1 Pt 2:678-81.
4. Garhofer G, Bek T, Boehm AG, Gherghel D, Grunwald J, Jeppesen P, Kergoat H, Kotliar K, Lanzl I, Lovasik JV, Nagel E, Vilser W, Orgul S, Schmetterer L, Ocular Blood Flow Research A. Use of the retinal vessel analyzer in ocular blood flow research. Acta Ophthalmol. 2010;88(7):717-22.
5. Heitmar R, Blann AD, Cubbidge RP, Lip GY, Gherghel D. Continuous retinal vessel diameter measurements: the future in retinal vessel assessment? Invest Ophthalmol Vis Sci. 2010;51(11):5833-9.
6. Hubbard LD, Brothers RJ, King WN, Clegg LX, Klein R, Cooper LS, Sharrett AR, Davis MD, Cai J. Methods for evaluation of retinal microvascular abnormalities associated with hypertension/sclerosis in the Atherosclerosis Risk in Communities Study. Ophthalmology. 1999;106(12):2269-80.
